# Behavioural asymmetries on the greyscales task: The influence of native reading direction

**DOI:** 10.1007/s40167-014-0019-3

**Published:** 2014-10-12

**Authors:** Trista E. Friedrich, Lorin J. Elias

**Affiliations:** Department of Psychology, University of Saskatchewan, 9 Campus Drive, Saskatoon, SK S7N 5A5 Canada

**Keywords:** Pseudoneglect, Perceptual asymmetries, Response bias, Reading direction, Spatial cognition, Cultural neuroscience

## Abstract

Reliable leftward attentional and perceptual biases demonstrated in a variety of visuospatial tasks have been found to deviate from the left in research examining the influence of scanning habits. The aim of the current research was to examine the influence of native script direction on pseudoneglect during the greyscales task in a representative sample of native right-to-left readers. Fifty-four native left-to-right readers and 43 right-to-left readers completed the greyscales task, which required judging the darker of two left–right mirrored brightness gradients. Native left-to-right readers demonstrated a left response bias on the greyscales task, whereas right-to-left readers failed to demonstrate a bias, however, both groups responded more quickly when making leftward choices. The research suggests that the strength of attentional biases are influenced by behavioural biases, such as scanning habits, and neural and anatomical asymmetries in the right parietal and frontal cortices. Thus, to improve the clinical utility of the greyscales task for diagnosing neglect, right-to-left readers should be examined to fully understand the normal range of biases displayed by neurologically healthy individuals.

A systematic asymmetry in spatial attention towards the left is reliably found in neurologically healthy participants, a phenomenon known as pseudoneglect as it mirrors rightward biases displayed by patients with right parietal lobe damage (Bowers and Heilman 1980; Jewell and McCourt 2000; Nicholls et al. 1999). A common tool used to investigate pseudoneglect and visuospatial attention is the greyscales task, which requires participants to judge equiluminant gradient stimulus pairs (Mattingley et al. 1994). When judging the gradient pair, Western samples of neurologically healthy participants reliability identify the luminance gradient with the pertinent feature located on the left side, irrespective of the directions requiring judgments of lighter, smaller and less numerous or darker, larger, and more numerous (Nicholls et al. 1999).

The leftward bias exhibited by neurologically healthy participants in a number of visuospatial tasks is less extreme compared to the rightward biases displayed by left spatial hemineglect patients, but both leftward and rightward biases have been found to involve the right posterior parietal cortex (PPC), the dorsolateral prefrontal cortex (PFC) and the superior longitudinal fasciculus (SLF) that interconnect the PFC and PPC (Bartolomeo et al. 2012; Corbetta et al. 2000; Foxe et al. 2003; De Schotten et al. 2011; Vallar 1998). Converging evidence from a large body of literature has suggested that preferential activation of the right hemisphere distributes attention to the left visual field and increases the salience of features located in the left hemispace (Bultitude and Davies 2006; Kinsbourne 1970; Loftus and Nicholls 2012; Ossandón et al. 2014; Nuthmann and Matthias 2013; Reuter-Lorenz et al. 1990).

In addition to the lateralized processing biases of the right hemisphere, several factors have been shown to influence the effect of pseudoneglect during the line bisection task including handedness, sex, assigned hand use, line length, line position and cueing; however, scanning direction, the direction which scanning is initiated, has been identified as the largest moderator of performance (Jewell and McCourt 2000). Research examining the manipulation of scanning direction during line bisection has identified that participants error towards the side that scanning was initiated (Brodie and Pettigrew 1996; Chokron et al. 1998; Halligan et al. 1991). Furthermore, learned behavior such as scanning habits, developed from reading direction, have been proposed to influence scanning strategies and the resulting outcome of perceptual asymmetries (Abed 1991; Manning et al. 1990). Compared to native left-to-right readers who reliably display a leftward bias, right-to-left readers have been found to display a central or rightward bias in several visuospatial tasks (Abed 1991; Chokron et al. 2009; Fagard and Dahmen 2003; Heath et al. 2005; Morikawa and McBeath 1992; Tse and Cavanagh 2000), including line bisection tasks (Chokron et al. 1997; Chokron and Imbert 1993; Chokron and De Agostini 1995); aesthetic preference tasks (Chokron and De Agostini 2000; Friedrich et al. 2014; Ishii et al. 2011; Nachson et al. 1999; Perez Gonzalez 2012), and lighting tasks (Smith and Elias 2013). Thus orientation of covert visual attention may be flexible (Foulsham et al. 2013) and influenced by visual scanning and scanning habits, leading to distinct performances on visuospatial tasks between native left-to-right and right-to-left readers. To the authors’ knowledge, limited research has used the greyscales task to examine pseudoneglect in native right-to-left readers, as the effect has primarily been observed in Western samples.

Research examining right-to-left readers performance on the greyscales task originated from Nicholls and Roberts (2002) and limited research has been conducted since. Nicholls and Roberts (2002) found a leftward bias for both left-to-right and right-to-left readers, concluding that attentional biases resulted from cues, rather than from scanning biases. However, Nicholls and Roberts (2002) used a small, homogenous sample of right-to-left readers (20 Hebrew participants), which may have influenced the authors’ null results. The right-to-left scanning tendency has been found to be stronger and more consistent in Arabic than Hebrew children (Kugelmass and Lieblich 1979), which may result from exposure of Hebrew readers to left-to-right notation in arithmetic, music, and the writing of some individual letters (Vaid and Singh 1989). Furthermore, the use of a small sample of participants who read Hebrew fails to adequately represent the right-to-left reading population. Hebrew is only the official language of Israel, and is spoken by approximately five million people worldwide, whereas other languages that are read from right-to-left such as Arabic, Persian, and Urdu is natively spoken in 59, 29, and six countries respectively and are collectively spoken by 343 million people worldwide (Lewis et al. 2013). Thus, it is difficult to determine the contribution of the Hebrew participant’s scanning habits to their performance on asymmetric visuospatial tasks.

Furthermore, Nicholls and Roberts (2002) did not specify whether Hebrew was the participant’s native language, which would also influence the perceptual biases found. Hebrew may have been the participants’ second language, as many Israelis learn Hebrew as their second language (Lewis et al. 2013) and Hebrew is only natively spoken by 49 % of the population (Central Bureau of Statistics Israel 2011). The reading direction of an individuals’ first language has been found to influence eye movement exploration (Abed 1991), leading to asymmetrical perception of space (Chokron and Imbert 1993), motion perception (Morikawa and McBeath 1992), and distribution of attention (Pollatsek et al. 1981). The unidentified native language of Nicholls and Robert’s (2002) participants questions the validity of the leftward bias found, as one’s mother tongue appears to influence perceptual biases.

The aim of the current research is to further investigate the influence of scanning habits on spatial attention biases using the greyscales task by examining a larger and more diverse sample of native right-to-left readers. It is predicted that left-to-right readers will reliably demonstrate a leftward attentional bias, consistent with previous literature examining Western populations (Mattingley et al. 1994; Nicholls et al. 1999). However, if non-directional stimuli are explored similar to ones native reading direction, as suggested by Abed (1991) and Chorkon and Imbert (1993), and if visual scanning direction influences orientation of attention, right-to-left readers are expected to demonstrate a weaker leftward, or central bias during the greyscales task.

## Methods

### Participants

A total of 97 participants participated in the study, 54 native left-to-right readers and 43 right-to-left readers (Table 1). All participants were fluent in English, and seventy-five of the participants, including the 43 right-to-left readers, were bilingual. Of the native left-to-right readers, 30 were male (*M* = 25.8 years, *SD* = 5.5 years), and of the native right-to-left readers 20 were male (*M* = 25.2 years, *SD* = 5.3 years). All procedures received ethical approval from the University of Saskatchewan Behavioural Research Ethics Board and all participants were recruited from the University of Saskatchewan through posters. All participants were paid for their participation in the study.

**Table 1:** Participant’s native language

LtoR reading languages	Number of participants	RtoL reading languages	Number of participants
English	22	Urdu	19
Hindi	14	Persian	12
Chinese	6	Arabic	8
Bengali	3	Aramaic	1
Korean	2	Hebrew	1
Malayalam	2	Pashto	1
Chichewa	1	Punjabi	1
Oriya	1		
Ukrainian	1		
Russian	1		
Vietnamese	1		

The native languages of left-to-right (LtoR) readers and the corresponding number of participants, as well as the native languages of right-to-left (RtoL) readers and the corresponding number of participants

### Greyscales task

**Fig. 1:**
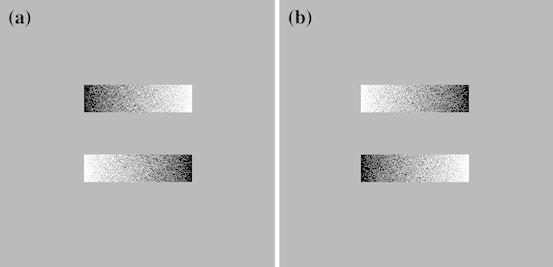
Sample stimulus pairs with opposite orientations from the greyscales task. Both *a* and *b* are 400 pixels long, but stimuli *a* is positioned with the upper stimulus *dark* on left and lower stimulus *dark* on right where as stimuli *b* is positioned with the upper stimulus *dark* on the right and lower stimulus *dark* on the left. A left response results from the participant choosing the stimulus with the *darker* feature on the left, irrespective of whether the stimulus is on the top or bottom

Participants simultaneously viewed two greyscales stimuli (Fig. 1) on a 17 inch CRT monitor. The horizontal midlines of the stimuli were aligned with the middle of the screen with a vertical distance of 100 pixels between the upper and lower stimulus. The stimuli were constructed via instructions from Nicholls et al. (1999). Two reversed luminance gradients that changed in brightness from one end to the other were outlined by a thin black outline and shown against a grey background. The stimuli measured 79 pixels high and changed in brightness over 80 increments, creating stimuli changing from black at one end to white at the other. The vertical position of the pixels within each increment was randomized to create a smooth change in brightness and create slight differences in the stimuli. The rectangles were presented as mirror reversals one on top of the other, but were equiluminant at a global level. The stimuli were presented in six different lengths: 320, 400, 480, 560, 640, and 720 pixels.

The task consisted of 96 trials that were presented in a pseudo-randomized order. The combinations of length (320, 400, 480, 560, 640, and 720 pixels) and stimulus orientation (upper stimulus dark on left/lower stimulus dark on right and vice versa) were repeated four times. Participants were seated 500 mm from the computer with the center of the monitor located along their midline. Participants were asked to determine which stimulus appeared darker overall, and responses were made using the numbers 8 and 2 on the number pad. The 8 represented the top rectangle, whereas the 2 represented the bottom rectangle. A simple button press was used to limit the amount of motor movement evoked by the task (McCourt and Olafson 1997). The participants’ responses were categorized based on which stimulus they selected as having the darker feature on the left or the right irrespective of whether it was on the top or bottom. A leftward response was indicated when the participant chose the stimulus with the darker feature on the left, irrespective of whether the stimulus was situated on the top or bottom, whereas a rightward response was indicated when the participant chose the stimulus with the darker feature on the right, irrespective of whether the stimulus was situated on the top or bottom. The response bias was calculated by subtracting the number of leftward responses from the number of rightward responses and could range from −96 to +96; hence a negative score indicated a leftward bias.

### Procedure

Participants were initially seated in a windowless room with overhead lighting and gave written consent to participate in the study. Prior to completing the greyscales task, participants completed a demographic questionnaire addressing sex, age, native language, visual or hearing impairments, and handedness and footedness (Elias et al. 1998).

## Results

**Fig. 2:**
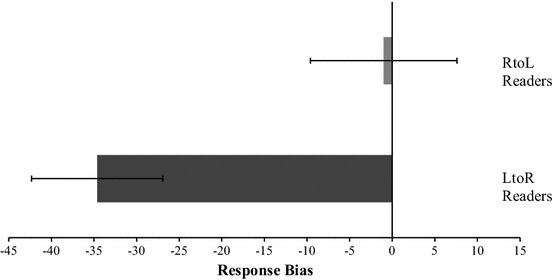
The mean response bias for the greyscales task demonstrated by native left-to-right (LtoR) and native right-to-left (RtoL) readers. A *negative score* indicates a preference for the darkest edge of the equiluminant gradient stimulus pair to be located on the left. *Error bars* represent standard error of the mean

The response bias was analyzed with a 2 (Reading direction [left-to-right, right-to-left]) × 2 (Sex [male, female]) repeated-measures ANOVA. There was a significant effect for reading direction, *F*(1, 93) = 8.489, *p* = .004, η_ρ_^2^ = .0894, but no significant effect of sex *F*(1, 93) = .026, *p* = .872, η_ρ_^2^ = .0003, and no interaction between the two factors, *F*(1, 93) = .049, *p* = .825, η_ρ_^2^ = .0005. The native left-to-right readers demonstrated a larger response bias (*M* = −34.6, *SD* = 37.67) compared to the right-to-left readers (*M* = −.99, *SD* = 42.06) (Fig. 2). One-sample *t* tests, compared to the test-value of zero, were conducted to evaluate whether a significant side bias was observed; left-to-right readers demonstrated a leftward response bias, *t*(53) = −5.097, *p* < .001, 95 % CI −48.28, −21.46, whereas right-to-left readers failed to demonstrate a significant bias *t*(42) = −.109, *p* = .914, 95 % CI −19.50, 17.45.

**Fig. 3:**
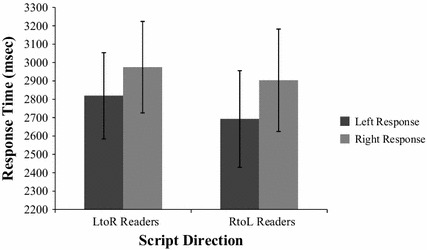
The mean reaction times for left-to-right (LtoR) and right-to-left (RtoL) readers left and right response choices. *Error bars* represent standard error of the mean

Additionally, the mean reaction time for left and right responses was analyzed with a 2 (Reading Direction [left-to-right, right-to-left]) × 2 (Response choice [right, left]) mixed-measures ANOVA (Fig. 3). There was no significant difference between the reaction time of left-to-right and right-to-left readers responses, *F*(1,95) = .079, *p* = .779, η_ρ_^2^ = .0008, and no interaction between the two factors, *F*(1, 95) = .091, *p* = .763, η_ρ_^2^ = .0009, however there was a significant difference between the reaction time for left and right choices, *F*(1, 95) = 4.029, *p* = .048, η_ρ_^2^ = .0424, with leftward responses (*M* = 2755.98, *SD* = 1736.18) occurring faster than rightward responses (*M* = 2939.12, *SD* = 1841.54).

## Discussion

In accord with a converging body of research examining left-to-right and right-to-left readers’ performance on a variety of visuospatial tasks, the reading groups demonstrated different bias strengths for the greyscales task. Consistent with previous research employing the greyscales task, native left-to-right readers in the present study demonstrated a statistically significant leftward bias (Mattingley et al. 2004; Mattingley et al. 1994; Nicholls et al. 1999). However, inconsistent with Nicholls and Roberts (2002) findings, native right-to-left readers failed to demonstrate a significant leftward bias. Thus, one’s native script direction appears to influence spatial attention. The sample of Hebrew readers examined by Nicholls and Roberts (2002) may have had increased exposure to left-to-right scanning order, leading to their null results. Hebrew children have been found to display weaker right-to-left tendencies compared to Arabic children (Kugelmass and Lieblich 1979), potentially resulting from the left-to-right notation of arithmetic, music, and writing of some individual letters in Hebrew (Vaid and Singh 1989). Increased exposure to left-to-right notation, especially if Hebrew was not Nicholls and Roberts (2002) participants’ native language, would have influenced the participants’ scanning tendencies and eye movement exploration, increasing the likelihood that a leftward bias will be observed (Abed 1991; Chokron and Imbert 1993).

The fact that participants with contrasting native reading directions performed differently on the greyscales task, demonstrates that scanning tendencies influence eye movement exploration and orientation of attention. Eye tracking experiments examining left-to-right readers have found reliable initial saccades at the beginning of visual exploration to the left side of a image followed by a longer and weaker bias to the right (Dickinson and Intraub 2009; Foulsham et al. 2013; Ossandón et al. 2014), even in the presence of a right-sided target (Nuthmann and Matthias 2013). However, these initial saccades to the left during scene viewing and line bisection task can be manipulated by the shape of a gaze-contingent window (Foulsham et al. 2013). Limited research has examined right-to-left readers’ visual exploration with eye tracking, however, Smith (2013) explored eye movements of right-to-left and left-to-right readers during a target finding task and observed that left-to-right readers identified targets the quickest when located in the upper left quadrant, whereas right-to-left readers had near identical identification times for targets located in the upper-right and upper-left quadrants. Thus native scanning habits and manipulation of viewing contingencies appear to influence initial fixation and location of attention.

The influence of scanning habits on attention asymmetries observed in the current study is consistent with literature examining the influence of scanning habits on visuospatial tasks. Left-to-right and right-to-left readers have demonstrated significantly different perceptual biases in line bisection tasks (Chokron et al. 1997; Chokron and Imbert 1993; Chokron and De Agostini 1995); aesthetic preference tasks (Chokron and De Agostini 2000; Friedrich et al. 2014; Ishii et al. 2011; Nachson et al. 1999; Perez Gonzalez 2012), and lighting tasks (Smith and Elias 2013). In addition to natural scanning habits developed from reading direction, manipulation of scanning direction during line bisection tasks (Brodie and Pettigrew 1996), and face perception (Butler and Harvey 2006) have also been found to influence the direction and magnitude of the perceptual bias.

Visuospatial tasks are perceptual, however, the strategies used to complete visuospatial tasks influence orientation of attention across the visual field (Thomas et al. 2014). Pesudoneglect is often explained by the preferential activation of the right hemisphere that distributes attention to the left visual field and increases the salience of features located in the left hemispace—the activation-orienting hypothesis (Kinsbourne 1970). Manipulating orientation of attention with cues during visuospatial tasks systematically biases perception leading participants to commonly overestimate portions of space to which their attention is directed, and thus spatial distribution of attention and attentional asymmetries have been proposed to underlie and influence perceptual biases (Milner et al. 1992; Nichelli et al. 1989; Niemeier et al. 2008; Reuter-Lorenz et al. 1990). Furthermore, orientation influences perceptual biases in right unilateral spatial neglect patients, as cues located on the left of visual spatial tasks annul rightward displacement (Nichelli et al. 1989). Specifically, the typical leftward response bias observed during the greyscales task is reversed by attentional cues, and the leftward response bias is insensitive to changes in the presentation of the stimuli (Nicholls et al. 2004; Nicholls et al. 2008). These converging results have lead researchers to suggest that the greyscales task is a robust and sensitive measure to attentional biases, which appear, based on the findings in the current experiment, influenced by scanning habits and reading direction.

The underlying neurological mechanisms responsible for the increased salience of features located in the left hemispace is currently debated (De Schotten et al. 2011; Ossandón et al. 2014; Reuter-Lorenz et al. 1990). However, right-to-left scanning habits developed from reading direction appears to interact with spatial attention that is oriented to the left hemispace, resulting in no perceptual bias. Thus left-to-right scanning habits developed from reading direction strengthen the lateralized bias whereas right-to-left scanning habits weaken the bias. This pattern is evident in the current study as both reading groups responded faster to leftward choices resulting from increased attention to the left hemifield, but the salience of stimulus with the darkest feature located on the left was weakened when participants’ native language read from right-to-left. Hence, the results of the current study lead us to argue that the strength of perceptual biases is influenced by behavioural biases, such as scanning habits, and neural and anatomical asymmetries in the right parietal and frontal cortices (Szczepanski and Kastner 2013; De Schotten et al. 2011).

To further isolate the influence of reading direction and scanning habits on perceptual biases researchers should examine homogenous sample groups to ensure that cultural differences are not contributing to the different response biases found. Ideally, research examining left-to-right and right-to-left reading groups should examine groups that have extralinguistic (e.g. context of usage, direction of arithmetic and music) and linguistic similarities (e.g. phonology and grammar), as well as a common geographical location and cultural foundation to minimize the confounding effects of cultural influences on reading direction. Based on our findings, examining two samples with similar cultural values, but who also differ in reading direction may accentuate the differing response biases currently observed.

From a clinical perspective, understanding how perceptual asymmetries manifest, regardless of their underlying cause, is essential when studying behaviour as a marker of cognitive processes. Patients with right unilateral hemispheric damage have been found to demonstrate a stronger rightward bias during the greyscales task (Mattingley et al. 2004), leading researchers to suggest that the greyscales task is a highly sensitive and efficient tool for assessing pathological perceptual and attentional biases (Mattingley et al. 1994). However, the altered response bias for right-to-left readers compared to left-to-right readers questions the strength and reliability of the leftward bias displayed by healthy participants, as the leftward bias is not consistent and is affected by learned cultural factors such as reading direction. Thus, clinicians should exhibit caution when using abnormal performance on the greyscales task to aid diagnosis of neglect, specifically when the patient’s native language reads from right-to-left. Furthermore, research should examine right-to-left reading populations to determine the normal range of bias scores for right-to-left readers to increase the clinical utility of the greyscales task.
